# Dual-port laparoscopic myomectomy: a balanced yet potentially more optimal surgical approach

**DOI:** 10.3389/fmed.2025.1617194

**Published:** 2025-07-08

**Authors:** Ying Liu, Qiang Zhang, Biao Huang, Xin Li, Tianjiao Liu, Lijuan Xu, Xiaoyan Liao, Jianmei Liao, Wei Cheng, Hui Wang, Juan Huang, Tenglan Wu, Yan Liu, Jie Yu, Yonghong Lin, Xiaoqin Gan

**Affiliations:** ^1^Department of Gynecology and Obstetrics, Chengdu Women’s and Children’s Central Hospital, School of Medicine, University of Electronic Science and Technology of China, Chengdu, China; ^2^Bazhong Maternal and Child Health Hospital, Bazhong, China; ^3^Department of Obstetrics and Gynecology, Women and Children’s Hospital of Chongqing Medical University, Chongqing, China; ^4^NHC Key Laboratory of Birth Defects and Reproductive Health, Chongqing, China; ^5^Key Laboratory of Birth Defects and Related Diseases of Women and Children, Ministry of Education, West China Second Hospital, Sichuan University, Chengdu, China

**Keywords:** dual-port laparoscopy, single-incision laparoscopy, myomectomy, minimally invasive surgery, perioperative outcomes

## Abstract

**Objective:**

This study aimed to compare the perioperative outcomes of single-incision laparoscopic surgery (SILS) and dual-port laparoscopic myomectomy in patients with solitary uterine fibroids.

**Methods:**

This retrospective observational study included 162 patients who underwent laparoscopic myomectomy for solitary fibroids from January 2022 to December 2023 at a single tertiary center. Patients were divided into a SILS group (*n* = 77) and a dual-port group (*n* = 85). Perioperative outcomes—including operative time, intraoperative blood loss, analgesic use, hospital stay, and cosmetic results—were compared between the groups. Multivariate linear and logistic regression analyses were conducted to identify factors associated with surgical complexity and recovery.

**Results:**

The dual-port group had significantly lower intraoperative blood loss (41.71 ± 65.37 mL vs. 89.55 ± 93.70 mL, *p* < 0.001), lower rates of postoperative analgesic use (24.7% vs. 40.3%, *p* = 0.034), and shorter hospital stays (1.07 ± 0.30 vs. 1.30 ± 0.65 days, *p* = 0.005) compared to the SILS group. Fibroid size and procedure time were independent predictors of increased bleeding. Posterior wall fibroids were significantly associated with postoperative analgesic use. Delayed discharge was more common in patients with larger fibroids and those requiring postoperative analgesia. Cosmetic outcomes in the dual-port group remained favorable despite the auxiliary incision.

**Conclusion:**

Dual-port laparoscopic myomectomy is a feasible and potentially more effective alternative to single-incision surgery, offering better ergonomic access, improved perioperative outcomes, and excellent cosmetic results. This approach may be especially advantageous when addressing large or posteriorly located fibroids. Individualized surgical planning remains essential to optimize outcomes in minimally invasive myomectomy.

## Background

Uterine fibroids (leiomyomas) are the most prevalent benign tumors among women of reproductive age, affecting up to 70% of this population (1, 2). While many cases are asymptomatic, a substantial proportion of women experience clinical symptoms such as heavy menstrual bleeding, pelvic pain, urinary frequency, and infertility (3, 4). For women who wish to preserve fertility or retain the uterus for other reasons, myomectomy remains the preferred surgical intervention. The evolution of minimally invasive surgical techniques has led to laparoscopic myomectomy becoming the gold standard, offering advantages including less postoperative pain, faster recovery, lower complication rates, and better cosmetic outcomes compared to open surgery (5–7).

In recent years, single-incision laparoscopic surgery (SILS) has gained traction due to its superior aesthetic results and potential to further reduce postoperative discomfort (8). However, despite its cosmetic appeal, SILS presents notable technical challenges. These include restricted instrument triangulation, limited maneuverability, and a higher likelihood of external and internal instrument collisions (9, 10). Such constraints may increase operative time, intraoperative blood loss, and surgeon fatigue, especially in procedures requiring complex dissection or suturing, such as myomectomy for larger or deeply embedded fibroids (11, 12).

To overcome these limitations, dual-port laparoscopic surgery has emerged as a modified minimally invasive approach (13). By introducing a small auxiliary port—typically only 5 mm in diameter—this technique preserves much of the cosmetic advantage of SILS while enhancing surgical ergonomics and precision. The additional port enables the formation of a triangular working space, improving instrument mobility and facilitating more efficient dissection and suturing (14, 15). Thus, dual-port laparoscopy is proposed as a balanced alternative, potentially optimizing both operative efficiency and patient satisfaction.

Despite the theoretical advantages, comparative data on dual-port vs. single-incision laparoscopic myomectomy remain limited, especially in cases involving solitary fibroids. Furthermore, the extent to which operative variables—such as fibroid size, location, and pelvic adhesions—impact outcomes across surgical techniques is poorly defined. A better understanding of these factors is essential to guide surgical planning and tailor treatment approaches according to patient-specific anatomical and clinical characteristics.

In this context, the present study aimed to compare the perioperative outcomes of single-incision and dual-port laparoscopic myomectomy in patients with solitary uterine fibroids. The analysis focused on surgical efficiency (operative time and blood loss), recovery-related indicators (pain management, gastrointestinal function, and hospital stay), and cosmetic considerations. By identifying key factors influencing surgical outcomes in each approach, this study seeks to provide practical insights into optimizing myomectomy strategies and enhancing individualized care in minimally invasive gynecologic surgery.

## Methods

### Study design and patient selection

This was a single-center, retrospective observational study conducted at Chengdu Women’s and Children’s Central Hospital, a tertiary referral center specializing in minimally invasive gynecologic surgery. The study period extended from January 2022 to December 2023. The protocol was approved by the institutional Ethics Committee of Chengdu Women’s and Children’s Central Hospital (No. 2022207), and informed consent was obtained from all participants prior to surgery. A total of 229 patients diagnosed with uterine fibroids and scheduled to undergo laparoscopic myomectomy were initially screened. The inclusion criteria were: (1) age between 18 and 50 years, (2) presence of a solitary uterine fibroid confirmed by preoperative ultrasound or MRI, and (3) planned laparoscopic myomectomy via either a single-incision or dual-port technique. Exclusion criteria included: (1) intraoperative concurrent procedures (e.g., ovarian cystectomy and hysteroscopic surgery), (2) multiple fibroids requiring extensive uterine reconstruction, (3) postoperative histopathological confirmation of adenomyosis or uterine malignancy, and (4) incomplete perioperative data. After applying the exclusion criteria, a total of 162 patients were included in the final analysis.

### Surgical procedures

All surgeries were performed by experienced gynecologic laparoscopists with more than 10 years of operative experience. Surgeons were categorized according to operative experience as either having ≥10 years or ≥20 years of independent laparoscopic surgical practice, and this variable was included in multivariate models to adjust for potential allocation bias. The choice of surgical approach—single-incision laparoscopy or dual-port laparoscopy—was based on surgeon preference and intraoperative feasibility.

#### Single-incision laparoscopic myomectomy (SILS group)

A 2–2.5 cm longitudinal incision was made in the umbilicus to insert a multichannel single-port device. A standard 10-mm laparoscope and conventional straight laparoscopic instruments were used for dissection, fibroid enucleation, and myometrial repair. Due to limited triangulation, instrument crossing and rotation were frequently required.

#### Dual-port laparoscopic myomectomy (dual-port group)

A 10-mm umbilical port was placed on the laparoscope, and an additional 5-mm auxiliary port was inserted in the lower left or right abdominal quadrant under direct visualization. This configuration enabled triangular instrument alignment, facilitating more ergonomic tissue manipulation and suturing. The remainder of the procedure followed the same steps as in the SILS group.

### Data collection and variables

Clinical and perioperative data were collected from electronic medical records. Baseline variables included age, body mass index (BMI), gravidity, parity, history of pelvic surgery, fibroid size and location (anterior vs. posterior wall), and the presence of pelvic adhesions (graded intraoperatively by the operating surgeon). Surgical outcomes included operative time (from skin incision to closure), intraoperative blood loss (estimated by suction volume minus irrigation fluid), conversion to multi-port or laparotomy, request for postoperative analgesics within 24 h, postoperative recovery indicators (time to first flatus, time to first oral intake, time to first ambulation, and time to first voiding), length of hospital stay, delayed discharge (defined as length of stay exceeding the institutional median by >1 day), and postoperative complications (including fever, infection, hematoma, or need for reoperation).

Complications were defined as adverse events occurring after surgery, including but not limited to wound infection, hemorrhage, thromboembolic episodes, and urinary tract-related issues. The severity of each complication was classified according to the modified Clavien–Dindo system, following the version adopted by our institution (Supplementary Table 1) (16).

### Pain and analgesia assessment

Postoperative pain was assessed using the Visual Analog Scale (VAS) at 3 and 6 h after surgery. Patients were offered non-steroidal anti-inflammatory drugs (NSAIDs) or opioids based on the severity of pain and their analgesic request. Analgesic use (yes/no) was recorded as a dichotomous variable. Photographic documentation of the abdominal wall was performed 1 week after surgery. Surgeons evaluated incision healing and size, with emphasis on the auxiliary port site in the dual-port group.

### Statistical analysis

All statistical analyses were performed using SPSS software (version XX.0, IBM Corp., Armonk, NY, United States). Continuous variables were expressed as mean ± standard deviation (SD) and compared using independent *t*-tests or Mann–Whitney *U*-tests, depending on their normality. Categorical variables were presented as counts and percentages and analyzed using chi-squared or Fisher’s exact tests as appropriate. Multivariate linear regression analyses were conducted to identify predictors of operative time and intraoperative blood loss. Multivariate logistic regression models were used to assess factors independently associated with postoperative analgesic use and delayed hospital discharge. Variables with a *p*-value of <0.10 in univariate analysis were entered into the multivariate models. Odds ratios (ORs) and beta coefficients (*β*) with 95% confidence intervals (CIs) were reported. A *p*-value of <0.05 was considered statistically significant.

## Results

**Figure 1:**
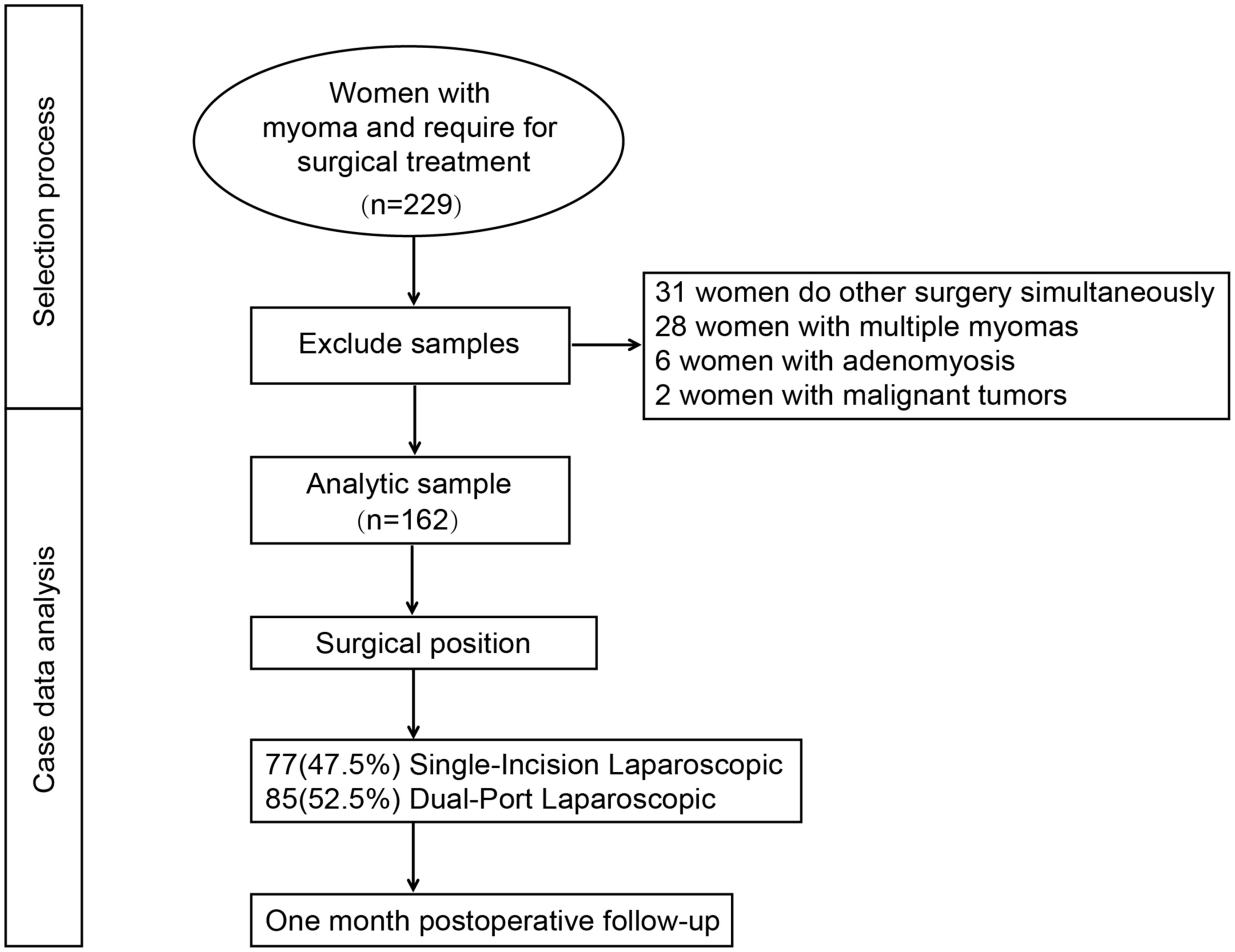
Selection process for this study.

A total of 229 patients with uterine fibroids were initially enrolled in this study (Figure 1). After excluding cases involving concurrent surgeries, multiple fibroids, postoperative pathological confirmation of adenomyosis, and malignant tumors, 162 patients were included in the final analysis. Among them, 77 patients (47.5%) underwent single-incision laparoscopic myomectomy, while 85 patients (52.5%) received dual-port laparoscopic myomectomy. The baseline demographic and clinical characteristics of the patients are summarized in Table 1. The mean age was 38.21 ± 7.26 years, and the mean BMI was 22.64 ± 3.08 kg/m^2^. A total of 76 patients (46.9%) had a history of pelvic surgery, including 62 (38.3%) with a prior cesarean section. No statistically significant differences were found between the two groups in terms of age, BMI, gestational history, or history of pelvic surgery (all *p* > 0.05).

**Table 1:** Description of the patients’ demographic characteristics and operation types.

Variables	Single-incision laparoscopic	Dual-port laparoscopic	*p*-value
Patients	*N* = 77	*N* = 85	
Age (year)	38.96 ± 7.63	37.54 ± 6.91	0.216^a^
BMI (kg/m^2^)	22.66 ± 2.94	22.62 ± 3.20	0.946^a^
History of pelvic surgery	34 (44.2%)	42 (49.4%)	0.503^b^
Maximum diameter of fibroid (cm)	6.59 ± 1.80	6.54 ± 1.64	0.855^a^
Gestation status			
Cesarean section	28 (36.4%)	34 (40.0%)	0.634^b^
Vaginal delivery	33 (42.8%)	26 (30.6%)	0.105^b^
Nulliparous	16 (20.8%)	25 (29.4%)	0.088^b^
Operative information			
Procedure time (min)	102.94 ± 32.05	97.55 ± 30.77	0.278^a^
Bleeding volume (mL)	89.55 ± 93.70	41.71 ± 65.37	<0.001^a^
Pelvic adhesions	36 (46.8%)	32 (37.6%)	0.436^b^
Fibroid location (posterior wall)	13 (16.9%)	30 (35.3%)	0.008^b^
Surgical conversion	5 (10.3%)	2 (1.9%)	0.259^c^
Postoperative information			
Time to first oral intake (h)	2.00 ± 1.01	2.08 ± 0.79	0.585^a^
Time to first ambulation	3.10 ± 1.57	3.00 ± 1.67	0.696^a^
Time to first urination (h)	3.25 ± 1.44	3.01 ± 1.76	0.367^a^
Time to first flatus (h)	12.54 ± 6.79	14.02 ± 6.62	0.177^a^
VAS score at 3 h	2.30 ± 1.29	2.28 ± 0.95	0.911^a^
VAS score at 6 h	2.04 ± 1.32	1.91 ± 0.74	0.525^a^
Request for analgesics	31 (40.3%)	21 (24.7%)	0.034^b^
Hospital stay	1.30 ± 0.65	1.07 ± 0.30	0.006^a^
Delayed discharge	17 (22.1%)	5 (5.9%)	0.003^b^
Infection	2 (3.4%)	1 (5.8%)	0.605^c^
Re-surgery	0 (0%)	0 (0%)	—

BMI: body mass index.

a Average and standard deviation. Student’s *t*-test.

b Number (percentage). Chi-squared test.

c Number (percentage). Fisher’s exact test.

Intraoperative parameters showed that the mean procedure time was comparable between the single-incision and dual-port groups (*p* = 0.278), whereas intraoperative bleeding volume was significantly higher in the single-incision group (*p* < 0.001). Additionally, the posterior wall fibroid location was more common in the dual-port group (*p* = 0.008), while the incidence of pelvic adhesions and surgical conversion rates did not differ significantly. In terms of postoperative recovery, the request for analgesics was significantly more frequent in the single-incision group (*p* = 0.034). Furthermore, the length of hospital stay was longer (*p* = 0.005), and the rate of delayed discharge was higher in the single-incision group (*p* = 0.003). No significant differences were observed between the two groups in terms of gastrointestinal recovery indicators, infection, or reoperation.

**Figure 2:**
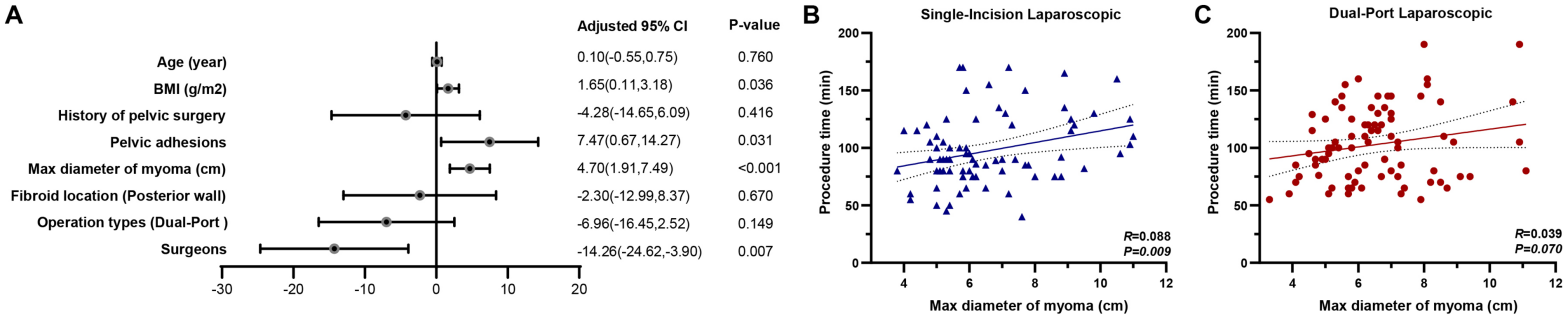
Impact of perioperative characteristics on procedure time. **(A)** In the multivariate linear regression analysis, larger fibroid size (*β* = 4.70, 95% CI: 1.91 to 7.49, *p* < 0.001), higher body mass index (BMI) (*β* = 1.65, 95% CI: 0.11 to 3.18, *p* = 0.036), and the presence of pelvic adhesions (*β* = 7.47, 95% CI: 0.67 to 14.27, *p* = 0.031) were significantly associated with prolonged operative time. In contrast, surgeries performed by more senior surgeons were associated with shorter operative durations (*β* = −14.26, 95% CI: −24.62 to −3.90, *p* = 0.007). **(B)** Subgroup analyses stratified by surgical approach revealed differential associations between fibroid size and operative time. A significant positive correlation was observed between the maximum diameter of fibroid and procedure time in the single-incision laparoscopic group (*R* = 0.088, *p* = 0.009). **(C)** However, this association was not statistically significant in the dual-port laparoscopic group (*R* = 0.039, *p* = 0.070), suggesting that dual-port laparoscopy may attenuate the operative time increase typically associated with larger fibroid size.

In the multivariate linear regression analysis (Figure 2A), larger fibroid size (*β* = 4.70, 95% CI: 1.91 to 7.49, *p* < 0.001), higher body mass index (BMI) (*β* = 1.65, 95% CI: 0.11 to 3.18, *p* = 0.036), and the presence of pelvic adhesions (*β* = 7.47, 95% CI: 0.67 to 14.27, *p* = 0.031) were significantly associated with prolonged operative time. Conversely, surgeries performed by more senior surgeons were associated with shorter operative durations (*β* = −14.26, 95% CI: −24.62 to −3.90, *p* = 0.007). Specifically, each 1 kg/m^2^ increase in BMI was associated with an approximately 1.7-min increase in procedure time; each level of severity of pelvic adhesions prolonged surgery by approximately 7.5 min; and each 1 cm increase in maximum fibroid diameter extended operative time by approximately 4.7 min. Other factors, including patient age, history of pelvic surgery, fibroid location (posterior wall), and surgical approach (single- vs. dual-port laparoscopy), were not independently associated with procedure duration.

Subgroup analyses stratified by surgical approach revealed differential associations between fibroid size and operative time. As illustrated in Figure 2B, a significant positive correlation was observed between the maximum diameter of fibroid and procedure time in the single-incision laparoscopic group (*R* = 0.088, *p* = 0.009). However, this association was not statistically significant in the dual-port laparoscopic group (*R* = 0.039, *p* = 0.070; Figure 2C), suggesting that dual-port laparoscopy may attenuate the operative time increase typically associated with larger fibroid size.

Factors associated with intraoperative blood loss were explored using multivariate linear regression analysis, as shown in Table 2. The results showed that operation type (*β* = 48.18, 95% CI: 23.15 to 73.22, *p* < 0.001) and procedure time (*β* = 0.75, 95% CI: 0.33 to 1.17, *p* = 0.001) were significantly associated with increased bleeding. Specifically, undergoing single-incision laparoscopy (reference: dual-port) was associated with an estimated 48.18 mL increase in blood loss, and each additional minute of procedure time resulted in approximately 0.75 mL of additional bleeding. Other variables, including age, BMI, surgical history, pelvic adhesions, fibroid size, fibroid location, and surgeon experience, were not significantly associated with bleeding volume (all *p* > 0.05).

**Table 2:** Association between operative bleeding and perioperative characteristics.

Variables	Beta	95% CI	*p*-value
*R*^2^ = 0.189			
Age (year)	0.35	(−1.37, 2.07)	0.688
BMI (g/m^2^)	−0.70	(−4.79, 3.39)	0.736
History of pelvic surgery	−12.52	(−39.75, 14.72)	0.365
Pelvic adhesions	15.27	(−2.81, 33.35)	0.097
Max diameter of fibroid (cm)	−6.22	(−13.80, 1.35)	0.107
Fibroid location (posterior wall)	−4.42	(−32.41, 23.58)	0.756
Operation types	−48.18	(−73.22, −23.15)	<0.001
Surgeons	2.32	(−25.46, 30.10)	0.869
Procedure time (min)	0.75	(0.33, 1.17)	0.001

BMI, body mass index.

The multivariate logistic regression analysis identifying predictors of postoperative analgesic medication use is summarized in Table 3. Among the variables examined, only fibroid location on the posterior uterine wall was significantly associated with increased odds of requiring postoperative analgesics (OR = 2.97, 95% CI: 1.31–6.77, *p* = 0.009). This indicates that patients with posterior wall fibroids were approximately three times more likely to require analgesic medication after surgery compared to those with fibroids in other locations. Other factors, including age, BMI, history of pelvic surgery, procedure time, intraoperative bleeding volume, presence of pelvic adhesions, fibroid size, surgical approach, and surgeon experience, were not significantly associated with postoperative analgesic use (*p* > 0.05 for all).

**Table 3:** Association between postoperative analgesic medication use and perioperative characteristics.

Variables	Exp (*B*)	95% CI	*p*-value
Age (year)	0.98	(0.93, 1.03)	0.390
BMI (g/m^2^)	1.05	(0.93, 1.19)	0.404
History of pelvic surgery	0.47	(0.21, 1.07)	0.071
Procedure time (min)	0.99	(0.98, 1.01)	0.246
Bleeding volume (mL)	1.00	(1.00, 1.01)	0.128
Pelvic adhesions	1.25	(0.74, 2.12)	0.408
Max diameter of fibroid (cm)	0.89	(0.71, 1.12)	0.335
Fibroid location (posterior wall)	2.97	(1.31, 6.77)	0.009
Operation types	0.48	(0.22, 1.05)	0.066
Surgeons	1.54	(0.66, 3.57)	0.318

BMI, body mass index.

Table 4 shows the results of the multivariate logistic regression analysis assessing factors associated with delayed hospital discharge. Two factors were found to be independently associated with an increased risk of delayed discharge. First, a larger maximum diameter of fibroid was significantly correlated with delayed discharge (OR = 1.43, 95% CI: 1.05–1.95, *p* = 0.025), indicating that each 1 cm increase in fibroid size was associated with a 43% increase in the odds of prolonged hospitalization. Second, postoperative use of analgesic medication was also significantly associated with delayed discharge (OR = 3.78, 95% CI: 1.21–11.76, *p* = 0.022), suggesting that patients who required postoperative analgesics were approximately four times more likely to experience delayed discharge. Other perioperative variables—including age, BMI, pelvic surgical history, pelvic adhesions, intraoperative blood loss, procedure time, fibroid location, operation type, and surgeon—were not significantly associated with discharge timing (*p* > 0.05 for all).

**Table 4:** Association between delayed discharge and perioperative characteristics.

Variables	Exp (*B*)	95% CI	*p*-value
Age (year)	1.06	(0.99, 1.14)	0.086
BMI (g/m^2^)	0.95	(0.78, 1.15)	0.583
History of pelvic surgery	2.39	(0.76, 7.55)	0.137
Procedure time (min)	1.00	(0.98, 1.02)	0.824
Bleeding volume (mL)	1.01	(1.00, 1.01)	0.094
Pelvic adhesions	0.99	(0.49, 2.00)	0.987
Max diameter of fibroid (cm)	1.43	(1.05, 1.95)	0.025
Fibroid location (posterior wall)	0.38	(0.09, 1.68)	0.203
Operation types	0.43	(0.13, 1.42)	0.165
Surgeons	1.41	(0.40, 4.96)	0.592
Analgesic medication use	3.78	(1.21, 11.76)	0.022

BMI, body mass index.

**Figure 3:**
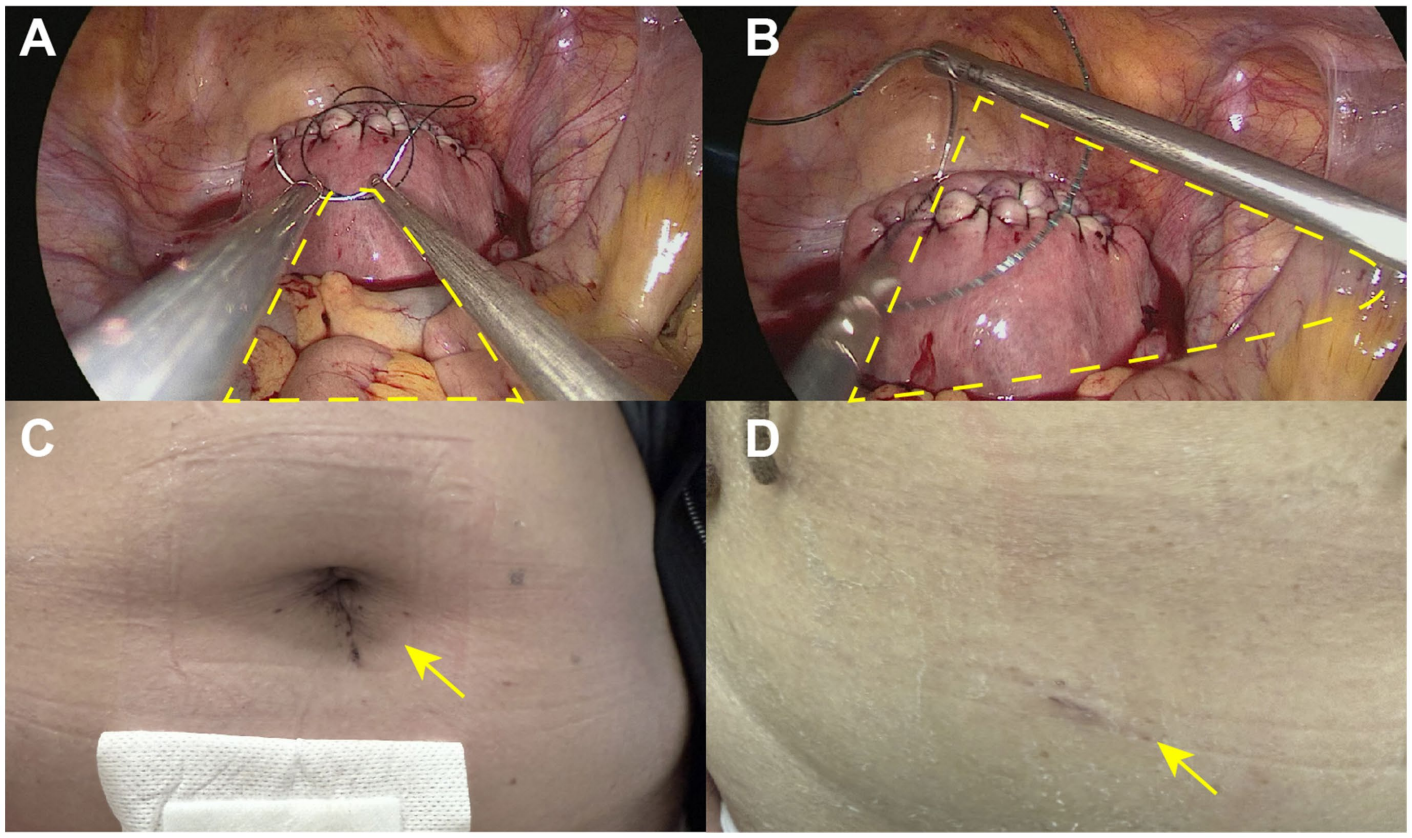
Instrument configuration and postoperative incision appearance between single-incision and dual-port laparoscopic approaches. **(A)** The instrument configuration in the single-incision laparoscopic approach, where all instruments are inserted through a single umbilical port. This setup results in limited triangulation and a narrower operative angle, which may restrict instrument maneuverability. **(B)** The dual-port laparoscopic approach, in which the use of two separate ports enables a wider triangular working space, thereby enhancing surgical precision and ease of operation. **(C,D)** The healing status of the umbilical and auxiliary port incisions 1 week after dual-port laparoscopic surgery.

Figure 3 compares the instrument configuration and postoperative incision appearance between single-incision and dual-port laparoscopic approaches. Figure 3A illustrates the instrument configuration in the single-incision laparoscopic approach, where all instruments are inserted through a single umbilical port. This setup results in limited triangulation and a narrower operative angle, which may restrict instrument maneuverability. In contrast, Figure 3B demonstrates the dual-port laparoscopic approach, in which the use of two separate ports enables a wider triangular working space, thereby enhancing surgical precision and ease of operation. Figures 3C,D show the healing status of the umbilical and auxiliary port incisions 1 week after dual-port laparoscopic surgery. The auxiliary incision is relatively small, indicating limited impact on surgical trauma and postoperative cosmetic appearance.

## Discussion

This study compared the perioperative outcomes of single-incision laparoscopic myomectomy (SILS) and dual-port laparoscopic myomectomy in patients with solitary uterine fibroids. The findings indicate that while both approaches are feasible and safe, the dual-port technique offers advantages in terms of reduced intraoperative blood loss, lower postoperative analgesic requirements, and shorter hospital stay, particularly in patients with larger fibroids or posterior wall lesions.

A key observation was that the single-incision approach was independently associated with increased intraoperative bleeding volume, even after adjusting for fibroid size and procedure time. This may be attributed to the ergonomic limitations of SILS, which include reduced triangulation, limited instrument range of motion, and suboptimal visualization in certain uterine positions (17, 18). In contrast, the dual-port technique—by allowing a more favorable triangular configuration—enhances the surgeon’s ability to dissect, cauterize, and suture more efficiently, thereby contributing to better hemostatic control. These findings are consistent with prior reports in the gynecologic surgery literature, which have demonstrated that limited access angles in SILS can compromise operative precision and increase blood loss, particularly in complex procedures such as myomectomy (19, 20).

Moreover, the analysis revealed that posterior wall fibroids were significantly associated with increased postoperative analgesic use. This likely reflects the greater surgical difficulty involved in accessing and excising fibroids located in the posterior uterus, especially through a single umbilical port (21, 22). Notably, the dual-port configuration seems to mitigate this difficulty, as suggested by the absence of a significant correlation between fibroid size and operative time in the dual-port group, in contrast to the SILS group, where a clear positive association was observed (23). These results highlight the utility of the auxiliary port in improving exposure and reducing surgeon stress during challenging dissections.

Another important outcome of this study was the association between larger fibroid diameter and delayed hospital discharge. While this finding may partly reflect increased surgical complexity and recovery burden, it also underscores the need to consider fibroid characteristics when selecting the most appropriate surgical approach. In addition, postoperative analgesic use independently predicted prolonged hospitalization, which is in line with previous studies showing that postoperative pain not only delays mobilization but also affects gastrointestinal function and discharge readiness (24). The reduced analgesic requirement and shorter hospital stay observed in the dual-port group further support the ergonomic and clinical benefits of this approach.

From a cosmetic perspective, dual-port laparoscopy preserved excellent aesthetic outcomes. Although an additional incision was required, the auxiliary port was only 5 mm in diameter and showed minimal scarring 1 week after surgery. This suggests that dual-port laparoscopy offers a practical balance between surgical performance and patient satisfaction (25, 26). While SILS has traditionally been promoted for its superior cosmetic results, our findings indicate that the trade-off in technical difficulty and perioperative burden may outweigh its limited aesthetic advantage, especially in cases involving larger or posterior fibroids.

The strengths of this study include its focused cohort of patients with solitary fibroids, reducing confounding by fibroid number or uterine distortion, and its detailed multivariate modeling accounting for a range of anatomical and clinical factors. Furthermore, all surgeries were performed by experienced laparoscopic surgeons, minimizing variability due to technical proficiency. Photographic documentation of incision healing also provided an objective measure of cosmetic outcomes, which are increasingly valued by patients undergoing minimally invasive gynecologic surgery.

Nevertheless, several limitations should be acknowledged. First, the retrospective and non-randomized nature of the study introduces the possibility of selection bias. Although baseline characteristics were well balanced, surgeon preference likely influenced the choice of surgical approach. Second, the study was conducted at a single institution, potentially limiting generalizability to other clinical settings with different patient populations or surgical practices. Third, the absence of long-term follow-up data on fertility preservation, symptom recurrence, and adhesion formation, as well as the inability to perform propensity score matching due to limited sample size, further restrict the generalizability and causal inference of our findings. Fourth, our findings may not be generalizable to women with multiple fibroids or complex pelvic anatomy, who were not included in this study. Future prospective or randomized controlled trials are needed to further confirm the results of this study.

## Conclusion

In summary, dual-port laparoscopic myomectomy appears to offer a balanced and potentially more optimal alternative to single-incision surgery, particularly in cases with larger fibroids or posterior uterine wall involvement. It combines the minimally invasive advantages of SILS with enhanced operative ergonomics and improved perioperative outcomes. These findings support the broader clinical adoption of dual-port techniques in appropriate patients and suggest that individualized surgical planning—taking into account fibroid characteristics, patient anatomy, and surgeon expertise—remains critical to optimizing outcomes in minimally invasive myomectomy.

## Data Availability

The original contributions presented in the study are included in the article/Supplementary material, further inquiries can be directed to the corresponding authors.
