# Supplementary material for: Dual-port laparoscopic myomectomy: a balanced yet potentially more optimal surgical approach
Source: Front Med (Lausanne). 2025 Jul 8;12:1617194. doi: 10.3389/fmed.2025.1617194 (PMC12279865; doi:10.3389/fmed.2025.1617194)
Supplement: Supplementary file 1 [file Table_1.docx]

**Supplementary Table 1 Modified classifications of Clavien-Dindo complications in our hospital**

| **Grades** | **Definition** |
| --- | --- |
| **Grade I** | Any deviation from the normal postoperative course without the need for pharmacological treatment or surgical, endoscopic and radiological interventions. Acceptable therapeutic regimens are drugs as antiemetics, antipyretics, analgesics, diuretics and electrolytes and physiotherapy. |
| **Grade II** | Requiring pharmacological treatment with drugs other than such allowed for grade I complications.  Poor wound healing treated at the bedside, re-catheterizating due to urinary retention, gastrointestinal decompression, intraoperative bleeding volume > 500 ml, blood transfusions and parenteral nutrition are also included. |
| **Grade III** | Requiring surgical, endoscopic, or radiological intervention. Intraoperative organ injury, postoperative venous thrombosis. |
| **Grade III-a** | Intervention not under general anesthesia. Intraoperative organ injury, postoperative venous thrombosis. |
| **Grade III-b** | Intervention under general anesthesia |
| **Grade IV** | Life-threatening complication (including CNS complications) * requiring IC/ICU-management |
| **Grade IV-a** | Single organ dysfunction (including dialysis) |
| **Grade IV-b** | Multi organ dysfunction |
| **Grade V** | Death of a patient |
| **Suffix 'd'** | If the patient suffers from a complication at the time of discharge. The suffix "d" (for 'disability") is added to the respective grade of complication. This label indicates the need for a follow-up to fully evaluate the complication. |

* Brain hemorrhage, ischemic stroke, subarrachnoidal bleeding, but excluding transient ischemic attacks (TIA); IC: Intermediate care; ICU: Intensive care unit
